# Where Did Life Begin? Testing Ideas in Prebiotic Analogue Conditions

**DOI:** 10.3390/life11020134

**Published:** 2021-02-10

**Authors:** David Deamer

**Affiliations:** Department of Biomolecular Engineering, University of California, Santa Cruz, CA 95060, USA; deamer@soe.ucsc.edu

**Keywords:** prebiotic analogue conditions, amphiphilic self-assembly, non-enzymatic polymerization

## Abstract

Publications related to the origin of life are mostly products of laboratory research and have the tacit assumption that the same reactions would have been possible on the early Earth some 4 billion years ago. Can this assumption be tested? We cannot go back in time, but we are able to venture out of the laboratory and perform experiments in natural conditions that are presumably analogous to the prebiotic environment. This brief review describes initial attempts to undertake such studies and some of the lessons we have learned.

## 1. Introduction

Most research on the origin of life is performed in the laboratory for obvious reasons. A laboratory setting is essential because the work is usually carried out not by an individual scientist but instead by teams of graduate students and post-doctoral associates who are supported by a grant for several years. Pure reagents can be ordered, pure water is prepared on site and advanced instrumentation like HPLC and mass spectrometry is available to analyze products. In general, the results from laboratory work are accepted as significant advances in understanding how life can begin. Examples include hundreds of publications from leading researchers who have established the fundamental underpinnings of several prebiotic processes. In all of these studies, there is a tacit assumption that the laboratory results have explanatory power related to similar reactions and processes that would have occurred in the prebiotic environment 4 billion years ago. This review aims to make the assumption explicit and to consider its validity. Research will be described from two laboratories that have accepted the challenge to carry out experiments in analogue sites.

## 2. Prebiotic Analogue Sites

**Figure 1:**
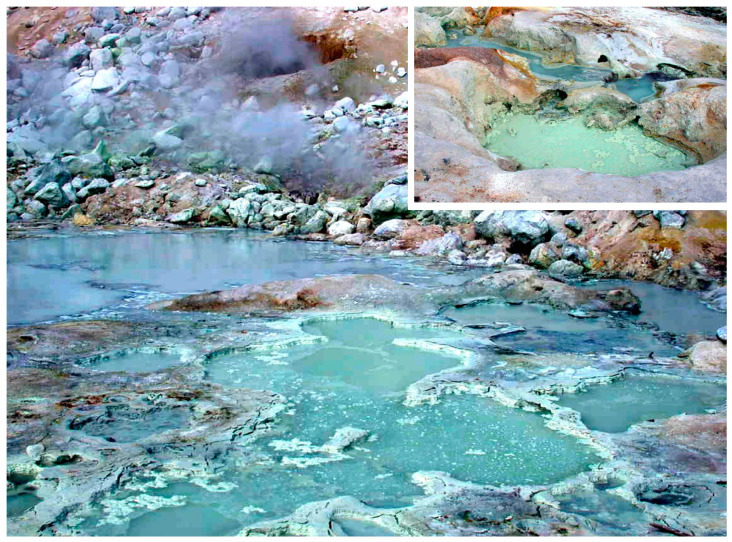
Bumpass Hell, a hydrothermal field on Mount Lassen in California. Inset shows a smaller, isolated pool nearby.

It is educational for origins of life researchers to visit one or more of the prebiotic analogue sites to be described here. Accessible examples include Yellowstone National Park in Wyoming and Hell’s Gate near Rotorua in New Zealand. Less accessible examples are remote sites like the volcanic areas of Kamchatka and Iceland, and the most difficult examples are hydrothermal vents that require diving in specialized submersible vehicles such as Alvin. When first viewing a hydrothermal site, a laboratory scientist is confronted by a daunting question: How could life begin in such conditions? There is no experimental control over standard parameters such as temperature, pH and ionic composition of the water. Furthermore, one imagines that the elevated temperatures and acidic or alkaline pH ranges would quickly destroy the materials commonly used in laboratory work. Proteins become denatured, enzyme activity is degraded and duplex structures required for nucleic acid functions melt into single stranded forms which may undergo further hydrolytic degradation. But after considering these seeming impossibilities, one comes back to the fact that certain microorganisms do thrive in extreme conditions, such as *Sulfolobus* in Yellowstone National Park hot springs and the microorganisms living in the porous mineral structures of black smokers and alkaline hydrothermal vents. It follows that hydrothermal conditions are not necessarily lethal and could even promote reactions that need heat to overcome the activation energy barrier. By analyzing certain minerals, Paul Knauth [1] estimated that the global temperature at the time of life’s origin was in the range of 55 to 85 degrees C. Fresh water hot springs on volcanic land masses are in this range, and so are alkaline hydrothermal vents. Figure 1 illustrates one such site called Bumpass Hell on Mount Lassen National Park in northern California.

Are such sites ideal analogues of prebiotic conditions? Hazen et al. [2] pointed out that clay minerals have evolved over time, and there is little doubt that the Great Oxidation Event changed the composition of clays and other minerals exposed to molecular oxygen. Russell and Ponce [3] proposed a set of ‘must have’ minerals related to life’s origin in hydrothermal vent conditions, but it is uncertain whether these were available on the prebiotic Earth. On the other hand, parameters such as temperature, evaporation, pH, dissolved salts and concentration of organic solutes are variables in today’s hot springs and are likely to be analogous to the same variables four billion years ago. For instance, the ionic composition of seawater and multiple hot springs has been described in [4], with a detailed discussion of implications for the origin of life. One example is shown in Table 1. The ionic solutes of a typical hot spring are much more diluted than seawater and it is reasonable to assume that they are analogous to prebiotic hot springs in this regard.

## 3. Results

### 3.1. Mount Mutnovsky, Kamchatka Russia

Our first attempt to carry out an experiment in a prebiotic analogue site was performed in 2004 on Mount Mutnovsky, an active volcano in Kamchatka, Russia. The local organizer was Vladimir Kompanichenko, a volcanologist who lives nearby in Khabarovsk, a city in southeastern Russia. The trip was sponsored by the NASA Astrobiology program and the group included two post-doctoral researchers from the Carnegie Institution of Washington, two graduate students in Chemistry at Stanford, and a photographer.

**Figure 2:**
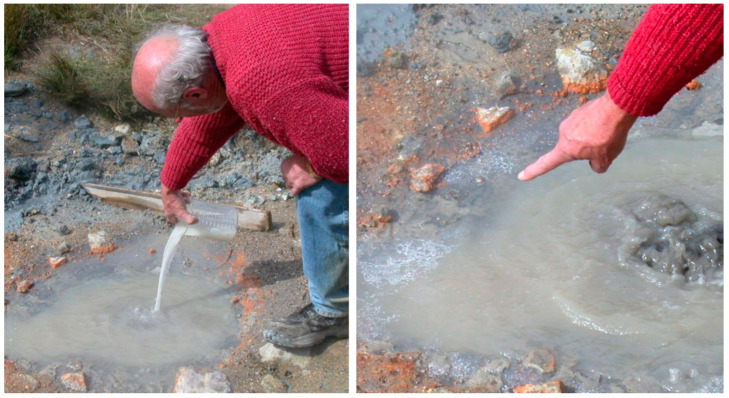
The author is shown adding a “prebiotic soup” to a boiling pool on Mount Mutnovsky, Kamchatka, Russia. The right hand panel shows the froth of myristic acid forming around the edge of the pool.

One of the experimental goals was to add the equivalent of a prebiotic soup to a small boiling pool having an estimated volume of ~10 L, then monitor the fate of the compounds over time (Figure 2). After adding the mixture, samples of the water were taken at time points measured in minutes, hours and days, then returned to our laboratory at UC Santa Cruz. We also took samples of the underlying clay layer for analysis. The mixture included four amino acids (glycine, L-alanine, L-aspartic acid, L-valine, 1 g each), four nucleobases (adenine, cytosine, guanine and uracil, 1 g each) sodium phosphate (3 g) glycerol (2 g) and myristic acid (1.5 g). We chose this mixture as a way to see how compounds with prebiotic relevance behave in hot spring conditions but also to monitor synthetic or degradation reactions that might occur. Myristic acid was chosen as a component of the mixture because previous studies revealed that it could assemble into membranous vesicles. Shorter chain lengths (10 and 12 carbons) also form membranes but require higher concentrations. The powdered mixture was first dissolved in ~ 1.0 L of hot water from the site, and the resulting milky solution was poured into the boiling center of the pond to start the experiment. The pool was acidic (pH 3.1) because of the sulfur dioxide dissolved in the water that becomes oxidized by atmospheric oxygen to sulfuric acid. Hot spring pools exposed to the anoxic early atmosphere would also be acidic from sulfurous acid produced by hydration of sulfur dioxide.

With one exception, the components disappeared from the solution in minutes to hours. They had adsorbed to the abundant clay particles that were suspended in the water by the boiling action and then settled back into the clay layer that lined the pool. Back in the laboratory, two gram samples of the clay were added to 10 mL of water and the pH was adjusted to an alkaline range by addition of sodium hydroxide. The adsorbed compounds were released as dissolved solutes in approximately the ratios at which they had been originally added [5]. The clay samples were analyzed by X-ray diffraction and turned out to be smectite mixed with kaolin.

The exception was myristic acid, a soap molecule, and 1.5 g in the mixture had been added to the pool. As shown in Figure 2, it was expected that a soap would separate from the aqueous phase and form a froth around the edge of the pool within minutes. A novel idea was suggested by this observation because during evaporation the soap would become a concentrated film on mineral surfaces along with other dissolved organic solutes. Cycles of dehydration and rehydration in such freshwater conditions would promote the assembly of membranous structures in which condensation reactions can produce polymers from dissolved monomers. Over the next 15 years, this concept has guided our research and led to several important insights. Perhaps most important is experimental evidence that polymerization reactions do occur within the drying film [6,7,8]. Upon rehydration, the polymers become encapsulated within membranous compartments to form vast populations of protocells that can undergo selection and evolution [9].

### 3.2. Self-Assembly of Amphiphiles in Yellowstone Hot Springs

A second experiment was to compare hot spring water and seawater in terms of their ability to support assembly of membranous vesicles. The question to be addressed concerned how much the pH and ionic composition of the hot spring water samples affected the ability of a mixture of amphiphiles to form membranous vesicles. The results were then compared directly to the much greater concentration of ions in seawater [10].

**Figure 3:**
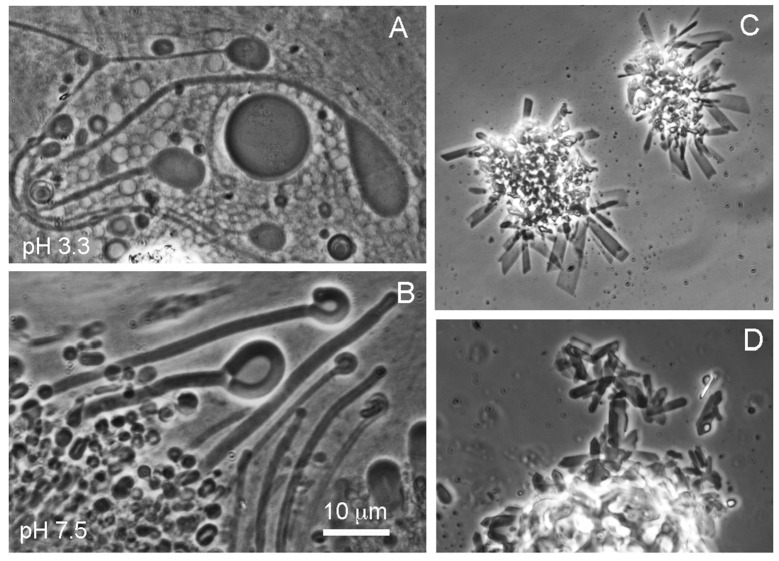
(**A**,**B**) show membranous structures that assemble from a mixture of dodecanoic acid and dodecanoyl monoglyceride in Yellowstone hot spring water at two different pH ranges [8]. (**C**,**D**) show the same compounds in seawater. Membranous structures cannot assemble, probably because crystals form when divalent calcium and magnesium cations bind to the carboxylate groups of the dodecanoic acid.

Water samples were collected from two different hot springs in Yellowstone National Park and returned to the laboratory for microscopy and analysis of ionic composition and concentration. It was clear that the low concentrations of ionic solutes in hot spring water permitted membranous vesicles to assemble at two pH ranges (Figure 3A,B). However, the ionic composition of seawater inhibited membrane assembly. Instead, the fatty acid crystallized, probably because the divalent ions present in seawater bound to the carboxylate groups and formed calcium and magnesium soaps (Figure 3C,D). It should be noted that this result was observed when a single fatty acid was present in the mixture. If a mixture of fatty acids having different chain lengths is used, membranous vesicles can assemble in artificial seawater exposed to simulated alkaline vent conditions [11].

### 3.3. Self-Assembly of Amphiphiles in Ladakh (India) Hot Springs

**Figure 4:**
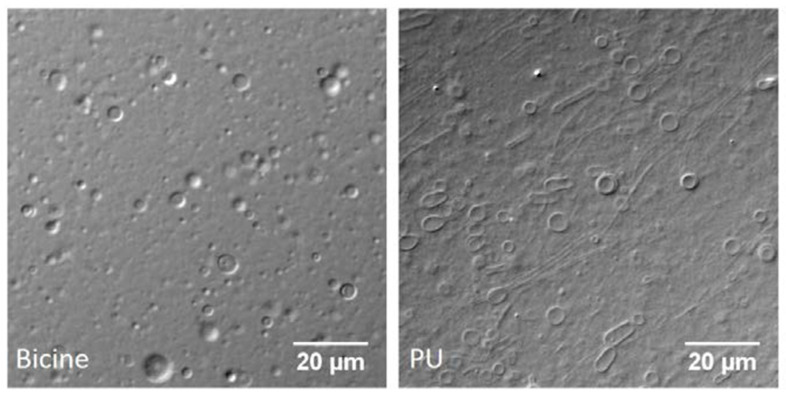
Joshi et al. [12] observed that a 2:1 ratio of decanoic acid (40 mM) with its monoglyceride (20 mM) readily formed membranous vesicles both in pure bicine buffer (200 mM, pH 8.5) and in a water sample from the Puga hot spring site in Ladakh, India.

A third field-based study was performed in hot spring sites of Ladakh in northern India [12]. In contrast to Bumpass Hell and Yellowstone hot springs, the pH is slightly alkaline, ranging from 7.4 to 8.7. Figure 4 shows that a mixture of decanoic acid (C10:0) and its monoglyceride readily formed vesicles both in bicine buffer pH 8.5 and in water samples from the Puga region of Ladakh. Experiments were also performed with water samples from two other Ladakh hot spring sites called Chumathang and Panamic to evaluate the generality of these results. Additionally, different combinations of other fatty acids like undecenoic acid (C11:1) and oleic acid (C18:1), along with their respective monoglyceride and alcohol derivatives, were tested in both laboratory conditions (with buffers) and using the hot spring water samples. The authors reported that only the combination of a fatty acid and its glycerol derivative could significantly form vesicles in all the hot spring water samples that were tested. These results were independent of the chain length and unsaturation of the fatty acid, which suggests that terrestrial geothermal fields might have incorporated ‘selection mechanisms’ that would have allowed certain types of reactions and mixtures to be selected for physicochemical constraints that prevailed at that site. Additionally, the mixed vesicles of fatty acid and its monglyceride were also moderately stable at high temperature and under wet-dry cycles, further bolstering their ability to serve as protocell compartments under hot spring conditions.

### 3.4. Non-Enzymatic Polymerization of Nucleotides in Hell’s Gate Hot Springs, New Zealand

**Figure 5:**
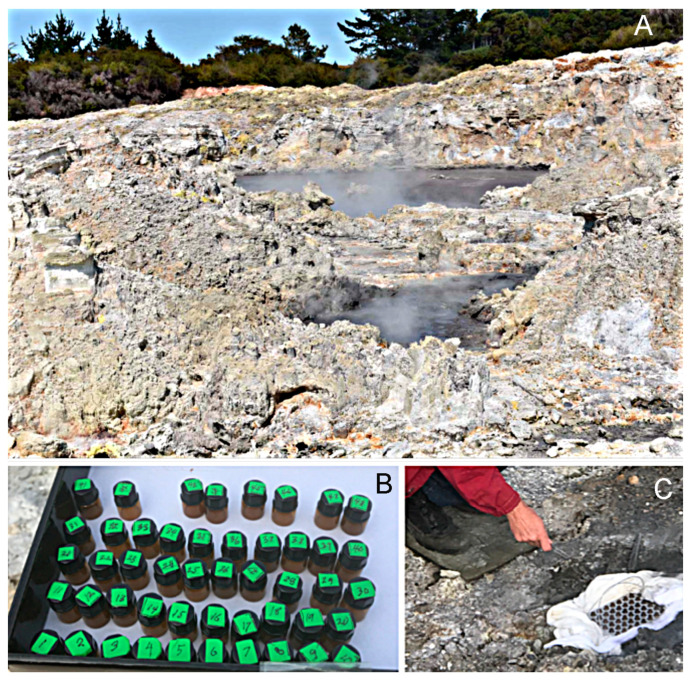
(**A**). Hell’s Gate hot spring site near Rotorua, New Zealand. (**B**) shows vials with samples prepared beforehand, and (**C**) shows the vials in place in a shallow pool of water. White fabric was placed around the container to prevent boiling water splashes from falling into the samples.

**Figure 6:**
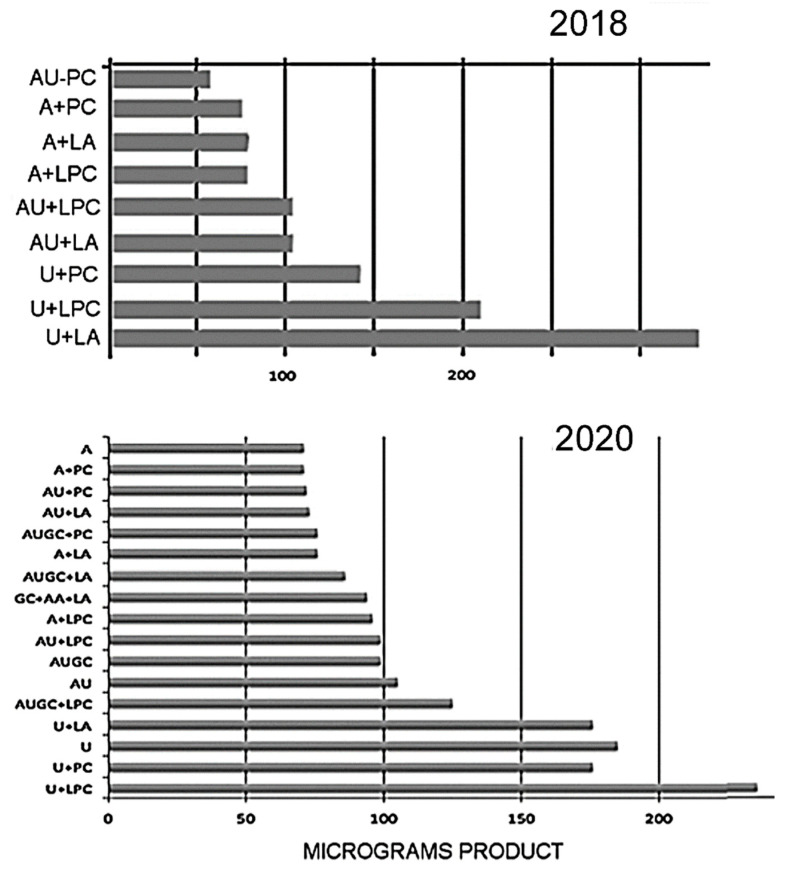
Polymer synthesis in nucleotide solutions exposed to three wet-dry cycles using hot spring water. Polymeric material was isolated by standard ethanol precipitation methods and yields were determined by Nanodrop spectrophotometry. Abbreviations: A, (AMP); U, (UMP); AU, (AMP+UMP, 1:1); GC, (GMP+CMP 1:1) AUGC, (all four mononucleotides in an equimolar mixture); LA, lauric acid mixed 1:1 with its monoglyceride; PC, phosphatidylcholine; LPC, lysophosphatidylcholine.

The most recent field studies were undertaken at a hot spring site called Hell’s Gate near Rotorua, New Zealand (Figure 5). The aim was to compare laboratory observations [6,7,8] that mononucleotides can polymerize when exposed to multiple wet-dry cycles at acidic pH ranges. Two series of preliminary experiments were performed in 2018 and 2020, with similar results (Figure 6). Each group of 4 vials contained a specific set of nucleotides, which in some samples were mixed with several kinds of amphiphilic molecules. The total concentration of all mononucleotides was 10 mM in the original solutions. The solutions (0.1 mL in each vial) were evaporated to dryness for transport, then at the hot spring site the vials were supported in an aluminum holder and placed in a shallow pool of water at 91 °C, pH 1.9. Hot spring water was filtered through a sterilizing Millipore filter, then 0.1 mL of the water was added to each vial to begin the experiment. The solution evaporated in a few minutes and the wet-dry cycle was repeated two more times at 30 min intervals. After returning the vials to the laboratory, products were analyzed by adding 0.1 mL of water to each vial, then 4 vials of each group were combined. Polymer products were precipitated in ethanol and the yields were determined by Nanodrop spectrophotometry.

Figure 6 shows a comparison of results from the experiments from which we learned three important lessons. First, if the experiment had failed there would be no polymeric products present in the mixture. Instead we found that significant amounts of polymers had been synthesized with UV spectra matching those expected from the mixed nucleotides. Yields ranged from 50 to 300 mg depending on which nucleotides were present in the mixture. Lower yields were observed if AMP was present, either by itself or in mixtures (50–100 μg total) while higher yields were obtained with UMP by itself (150–300 μg). Last, the presence of amphiphilic compounds had only a modest effect on the yields, increasing it two-fold in some experiments but not at all in others.

**Figure 7:**
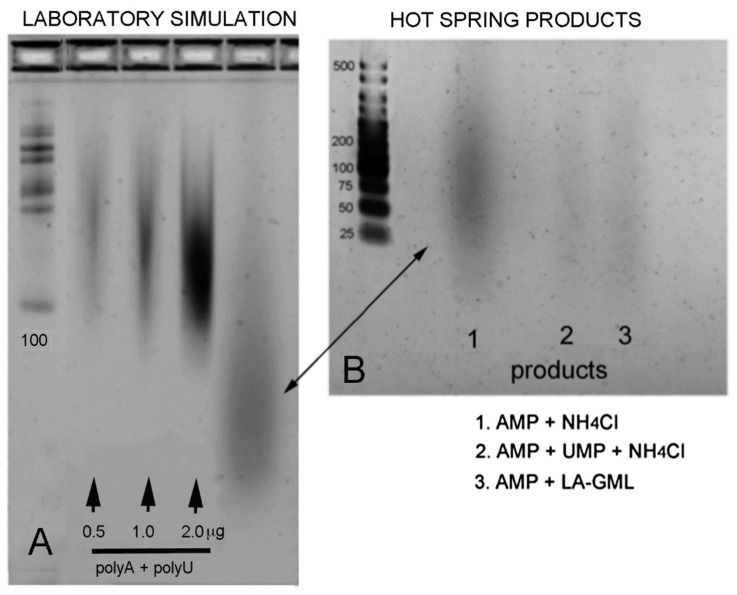
Polymeric products were compared by gel electrophoresis [4]. The ladder in (**A**) begins with 100 nt, and in (**B**) ranges from 25 to 500 nt. The standard control polymers in (**A**) were a 1:1 mixture of polyadenylic and polyuridylic acid which produced a blurred streak between 100 and 200 nt in length. Because they were a mixture of chain lengths, polymer products indicated by an arrow also produced a blurred streak ranging from <25 to >100 nt in length. Images were inverted for illustration purposes so that fluorescence appears dark on a light background. Figure reproduced from reference [4].

An additional indication that the polymers were composed of mononucleotides is that they moved during gel electrophoresis as though they were polyanions and were stained by the fluorescent dye ethidium bromide as expected for RNA-like polymers. Figure 7 shows two gels: one with polymers prepared in the laboratory and a second showing polymers prepared at Hell’s Gate in New Zealand.

The three center lanes in Figure 7A show positive controls consisting of microgram quantities of a 1:1 mixture of polyadenylic acid and polyuridylic acid with ethidium bromide present in the gel. This mixture is known to undergo extensive base pairing to form a double helical structure that is strongly stained by intercalating dyes. The lane just to the right is a polymer synthesized by wet-dry cycling of AMP and UMP in the laboratory. The polymers range from ~20 to over 100 nucleotides in length. Figure 7B shows polymers synthesized from AMP and UMP by cycling with acidic water in the Hell’s Gate hot spring. The arrow shows a range similar to that of the laboratory simulation. Both gels were stained with ethidium bromide and the images were inverted to increase contrast for illustration purposes.

## 4. Discussion

We have demonstrated that it is possible to test the assumptions of laboratory simulations in prebiotic analogues of hydrothermal fields and hot springs. In the future, laboratory simulations should also take into account what we have learned from ancient hot springs that existed 3.5 billion years ago [13]. One such example is the Pilbara Craton of Western Australia [14,15], which has preserved both ancient hot spring mineral composition and fossilized stromatolites. Of particular importance is the discovery of elements essential for life in the hydrothermal veins, including boron, zinc, manganese and potassium. With the exception of boron [16], these elements have not been incorporated in laboratory simulations of prebiotic chemical evolution. Their addition may provide further insights into how mineral solutes can affect reactions leading toward the origin of life.

The results described here show that both self-assembly of membranous compartments and polymerization reactions are feasible in low ionic strength hot spring water. The polymerization results must still be classified as preliminary, however, because the exact chemical structures of the polymers formed from ribonucleotides have not been elucidated. It is certain that they are composed of ribonucleotides because their UV spectra matches that of the reactants. What is uncertain is the kinds of linking bonds that are present. For instance, both 2′-5′ and 3′-5′ phosphodiester bonds are possible and the polymers are likely to contain both. It is also certain that they are polyanions resembling RNA because they readily migrate during gel electrophoresis. Furthermore, they are recognized by the enzymes used for end labeling with P-32-ATP [6]. Some of them are linear polyanions because they produce ionic current blockades when analyzed by nanopore methods [7].

Although the comparison of laboratory simulations with prebiotic analogue results was encouraging, there are certain limitations that should be made explicit. One concern is that conditions in the field today may not be fully analogous to the prebiotic Earth because atmospheric oxygen has altered the composition of the mineral surfaces exposed to hot spring water [2]. It is also possible that atmospheric oxygen can damage potential reactants and products, something that would not happen in the anoxic prebiotic atmosphere. However, the reactants we used are relatively resistant to oxidation, particularly for short exposures of hours, not years. A last variable to keep in mind is simply the size of the pools as discussed by Clark and Kolb [13]. For typical field experiments like those in Kamchatka, pools should range from half a liter to several liters in volume. Larger ponds would require hundreds of grams rather than gram quantities of reactants for a similar experiment. On the other hand, in order to do wet-dry cycling at a site such as Hell’s Gate, sample volumes must be less than a milliliter for evaporation to occur in a reasonable time frame.

In our experience, there are other hurdles that must be overcome. Many of the sites are in national parks and there is a fairly complicated permit process required to work there. After obtaining permits, logistical problems begin. A principal investigator will usually need to bring along a team of students and post-doctoral research associates. This means that visas must be obtained and housing arranged, as well as food and water for the actual field work. Equipment must be shipped, sometimes internationally, then transported to the research site. Then there are time constraints. In the laboratory, years can be spent on a research problem, but field work must usually be completed in weeks.

Finally, there is significant danger. Bumpass Hell on Mount Lassen is named after Kendall Bumpass, who was guiding a reporter to the newly discovered site in 1864 when he broke through a mineral crust into a boiling mudpot and burned his leg so badly it needed to be amputated. Another problem is that volcanoes emit potentially toxic gases like carbon dioxide, sulfur dioxide, hydrochloric acid and even hydrofluoric acid, so gas masks must be included in the equipment if the site is undergoing active volcanic activity. Despite the limitations and danger, if laboratory results can be confirmed by testing in prebiotic analogue conditions, it will make us more confident that further progress is possible as we address the question of life’s origin.

## Figures and Tables

**Table 1:** Ionic solutes of seawater and hot spring water from Yellowstone National Park.

Seawater		* Norris Geyser Basin T = 86 °C	
pH	8.1	pH	4.1
Na^+^	469 mM	Na^+^	13 mM
Cl^−^	546 mM	Cl^−^	17 mM
Ca^2+^	10 mM	Ca^2+^	0.120 mM
Mg^2+^	53 mM	Mg^2+^	0.026 mM
K^+^	10 mM	K^+^	1.4 mM
HCO_3_^−^	2 mM	HCO_3_^−^	0 mM
SO_4_^2−^	28 mM	SO_4_^2−^	1.3 mM

* Yellowstone National Park.
