# Addendum: Deamer, D. Where Did Life Begin? Testing Ideas in Prebiotic Analogue Conditions. *Life* 2021, *11*, 134

**DOI:** 10.3390/life11070613

**Published:** 2021-06-25

**Authors:** David Deamer

**Affiliations:** Department of Biomolecular Engineering, University of California, Santa Cruz, CA 95060, USA; deamer@soe.ucsc.edu

## Addendum

The author wishes to add the following information to the acknowledgements section of his paper published in *Life* [1].

The author thanks Kathleen Campbell and her students and staff for organizing the field work in New Zealand, and Bruce Damer for his collaborative efforts in the ongoing research. The author also thanks the staff at Hell’s Gate for their help and support in conducting the field work. The author greatly appreciated the assistance of UNSW graduate student Luke Steller in obtaining samples, and transportation to and from the site by Bryan Drake.

## References

[1] Deamer D. (2021). Where Did Life Begin? Testing Ideas in Prebiotic Analogue Conditions. Life.

